# Supplementary material for: Lowering the thermal noise barrier in functional brain mapping with magnetic resonance imaging
Source: Nat Commun. 2021 Aug 30;12:5181. doi: 10.1038/s41467-021-25431-8 (PMC8405721; doi:10.1038/s41467-021-25431-8)
Supplement: Supplementary file 1 — Supplementary Information [file 41467_2021_25431_MOESM1_ESM.pdf]

SUPPLEMENTARY MATERIAL

The supplemental information presented in this section falls into two categories**: I)** Supplementary Figs. 1 through 7, showing additional analyses and results based on the 0.8 mm isotropic resolution 7 Tesla data presented in the main body of the paper to supplement the conclusions reached from these 7T data; **II)** additional data sets and discussion demonstrating the wide applicability of NORDIC across field strengths, cortical regions, stimulation and/or task paradigms, and acquisition strategies.

I. Supplementary Figures based on the 7T data presented in the main body of the paper.

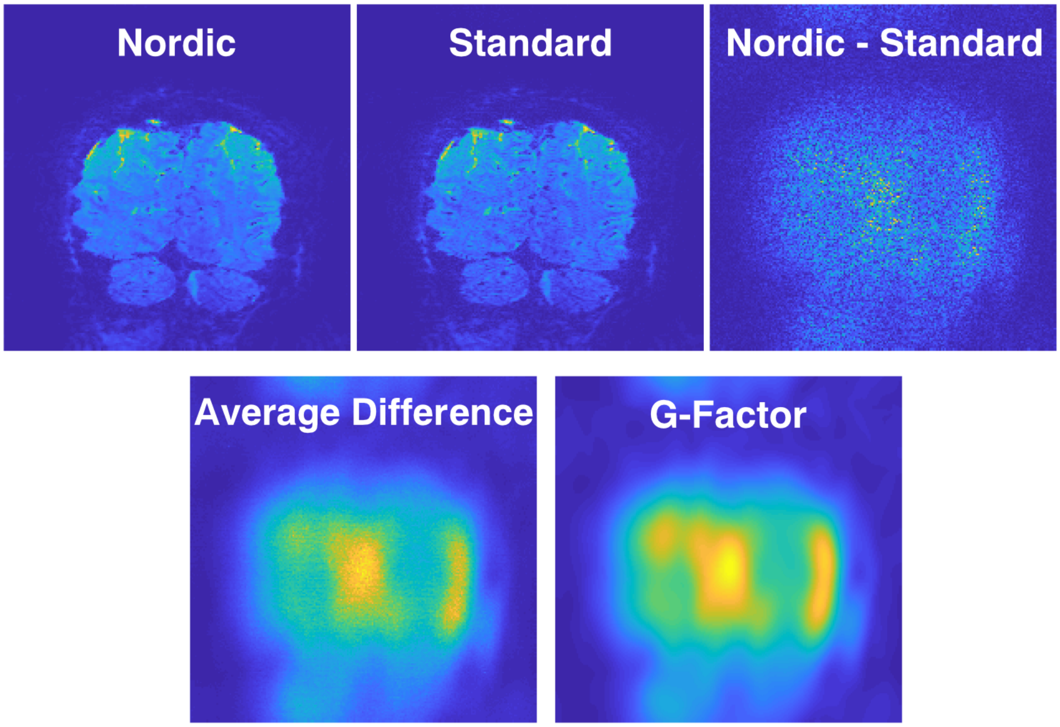

Supplementary Figure 1. Difference images obtained by subtracting Standard reconstructed image from the same image reconstructed with NORDIC. The top row shows data from a single slice and a single time point in the fMRI time series obtained from subject 1 with 0.8 mm isotropic resolution voxels and the block design target and surround retinotopic stimuli presented in the main text (Figs. 1 through 5). NORDIC and Standard reconstructed images of this slice are shown together with a difference between these reconstructions (top row, right most panel). Note how the structured noise reflects the G-factor (shown in the rightmost panel second row), which, incidentally, is comparable to the average of Nordic-Standard across all volumes. The lower row, left panel shows the same NORDIC minus Standard difference for the same slice but now averaged for all time points in the fMRI time series. Lower row right panel shows the g-factor map. These results demonstrate that there are no structures (e.g. edges) in the difference maps reflecting the features of the images they come from; such edge effects would be expected if there is blurring of the image by NORDIC Furthermore, when averaged over all data in the fMRI time series, the resulting image (lower row, left panel) looks like the g-factor map (lower row, right panel), as would be expected if NORDIC is removing the instrumental thermal noise amplified by the g-factor.

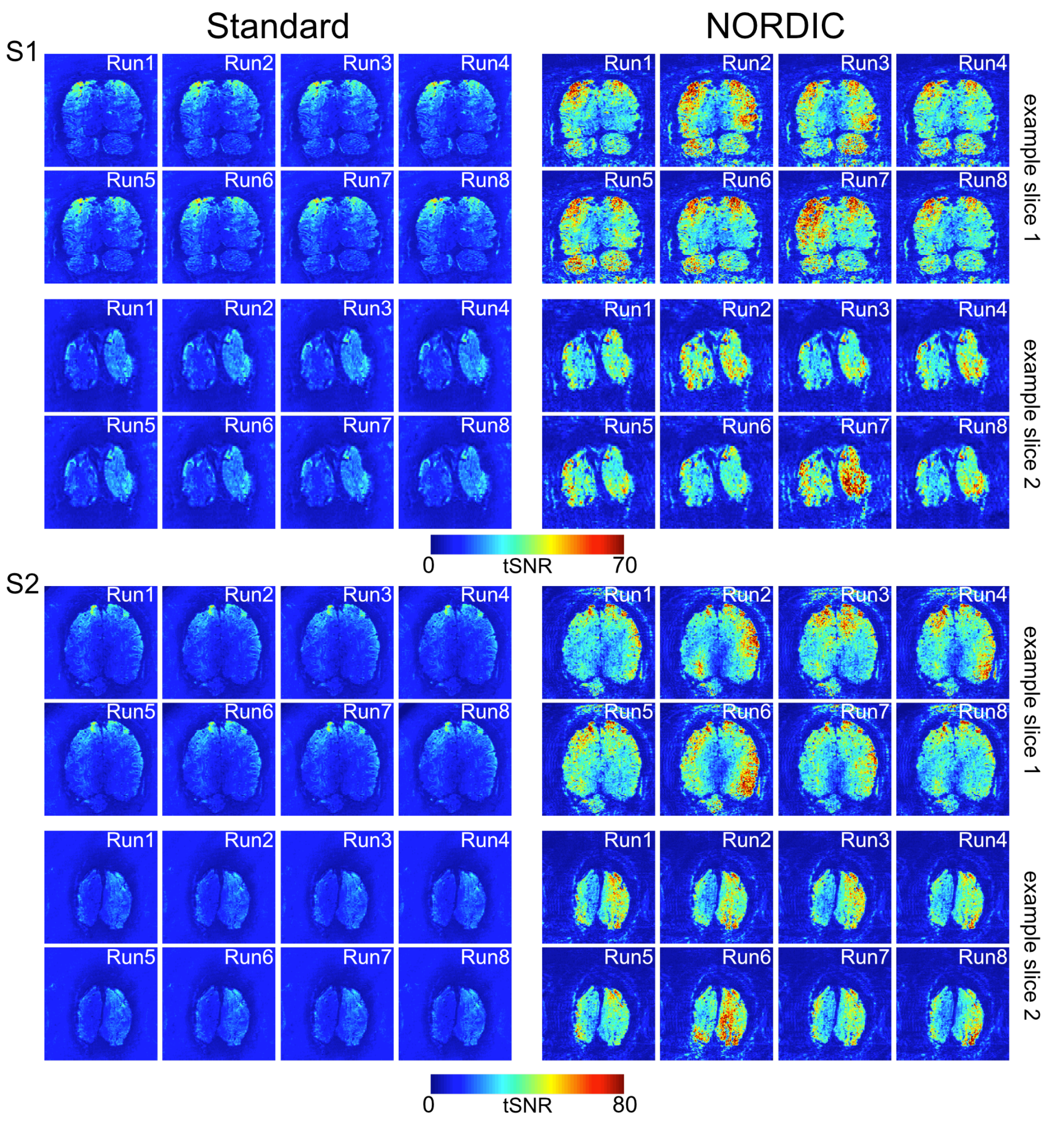

Supplementary Figure 2. Single runs temporal signal-to-noise ratio (tSNR) maps. tSNR maps of 2 exemplar slices for two subjects for all 8 different runs for Standard (left) and NORDIC (right) reconstructions. Slice 1, represents one of the anterior most slices in the covered volume; slice 2 is an occipital slice that includes a portion of V1.

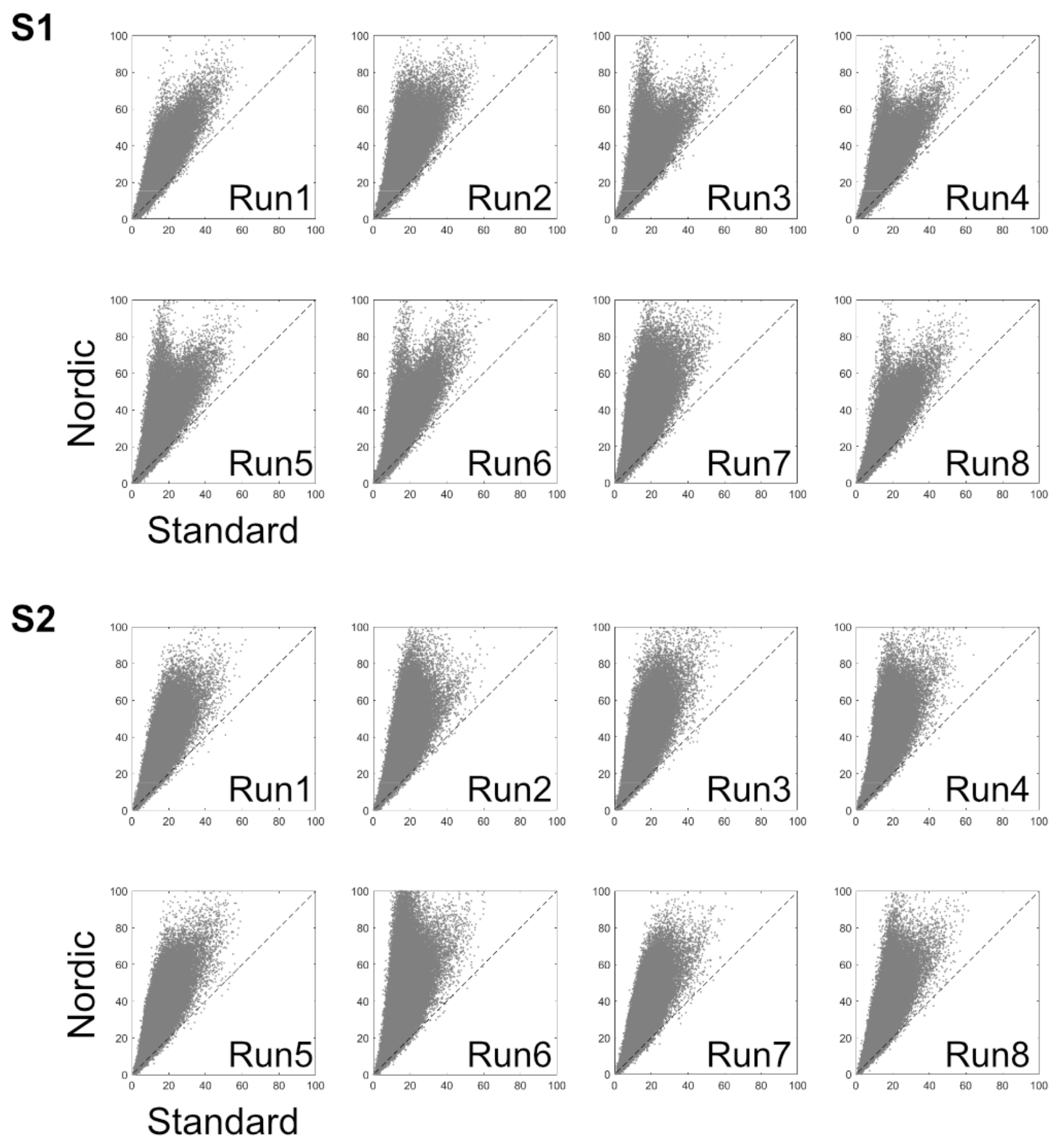

Supplementary Figure 3. Single run temporal signal-to-noise ratio (tSNR) scatter plots. Single run scatterplots of the temporal SNR of NORDIC vs Standard for all brain voxels for 2 representative subjects (S1 and S2, in Figure 2, main manuscript). Source data are provided as source Data file.

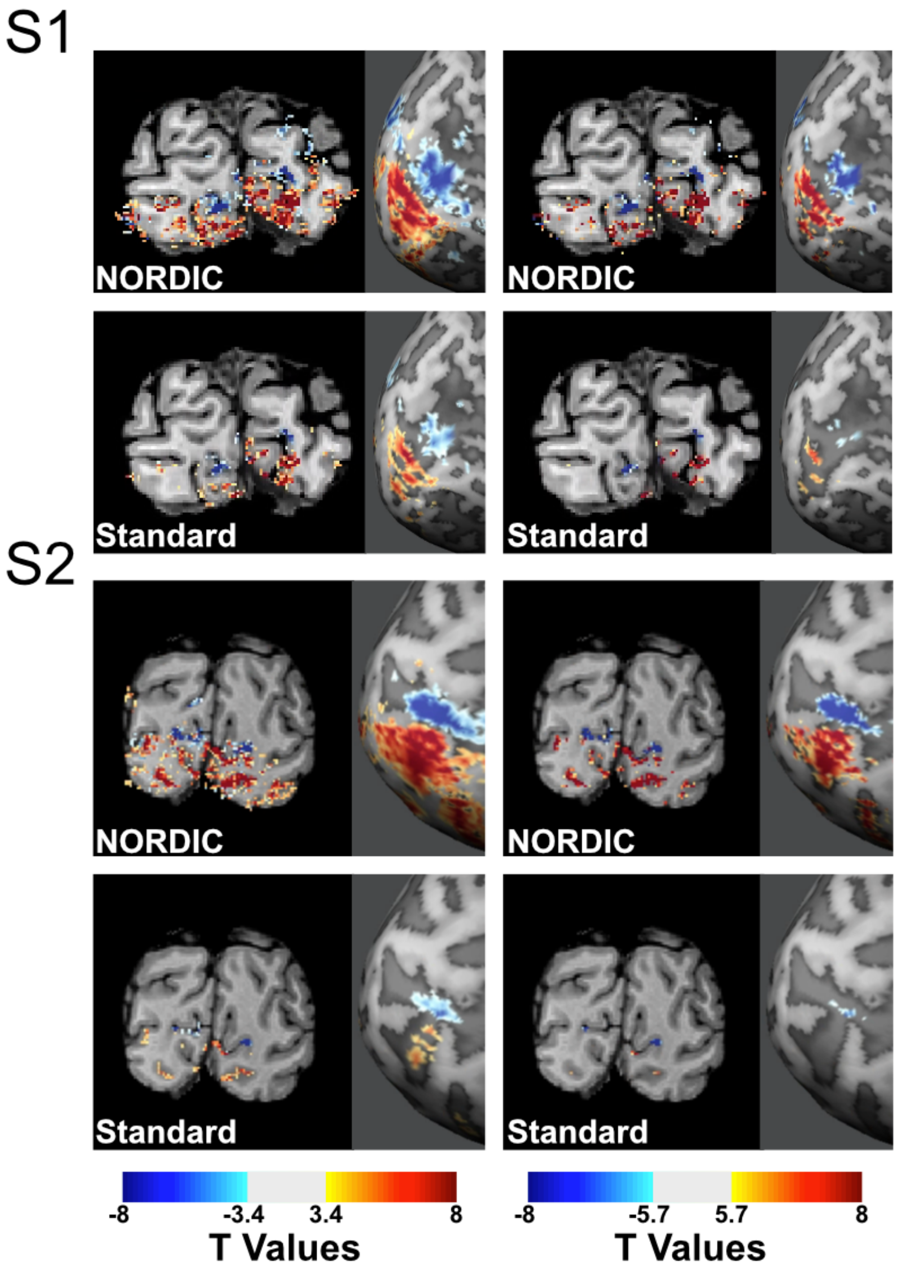

Supplementary Figure 4. NORDIC vs. Standard t-maps. Examples of NORDIC and Standard reconstructed t-maps superimposed onto T1 weighted anatomical images for two subjects (S1 and S2, in Figure2, main manuscript) obtained from a single ~2.5 min. For each subject, the images on the top row represent NORDIC reconstruction and the lower row the Standard reconstruction. The t-maps were computed by contrasting the activation elicited by the target (red) *versus* that elicited by the surround (blue) condition for a single representative run in volume space (left) and on inflated brains (right). The two different columns show different thresholds, specifically, $t \geq 3.4$ (left column) and $t \geq 5.7$ (right column); for the Standard reconstruction. For the Standard reconstruction. These t-values (from a 2-sided paired sample t-test) correspond to $p<0.01$ (uncorrected) and $p < 0.05$ (Bonferroni corrected).

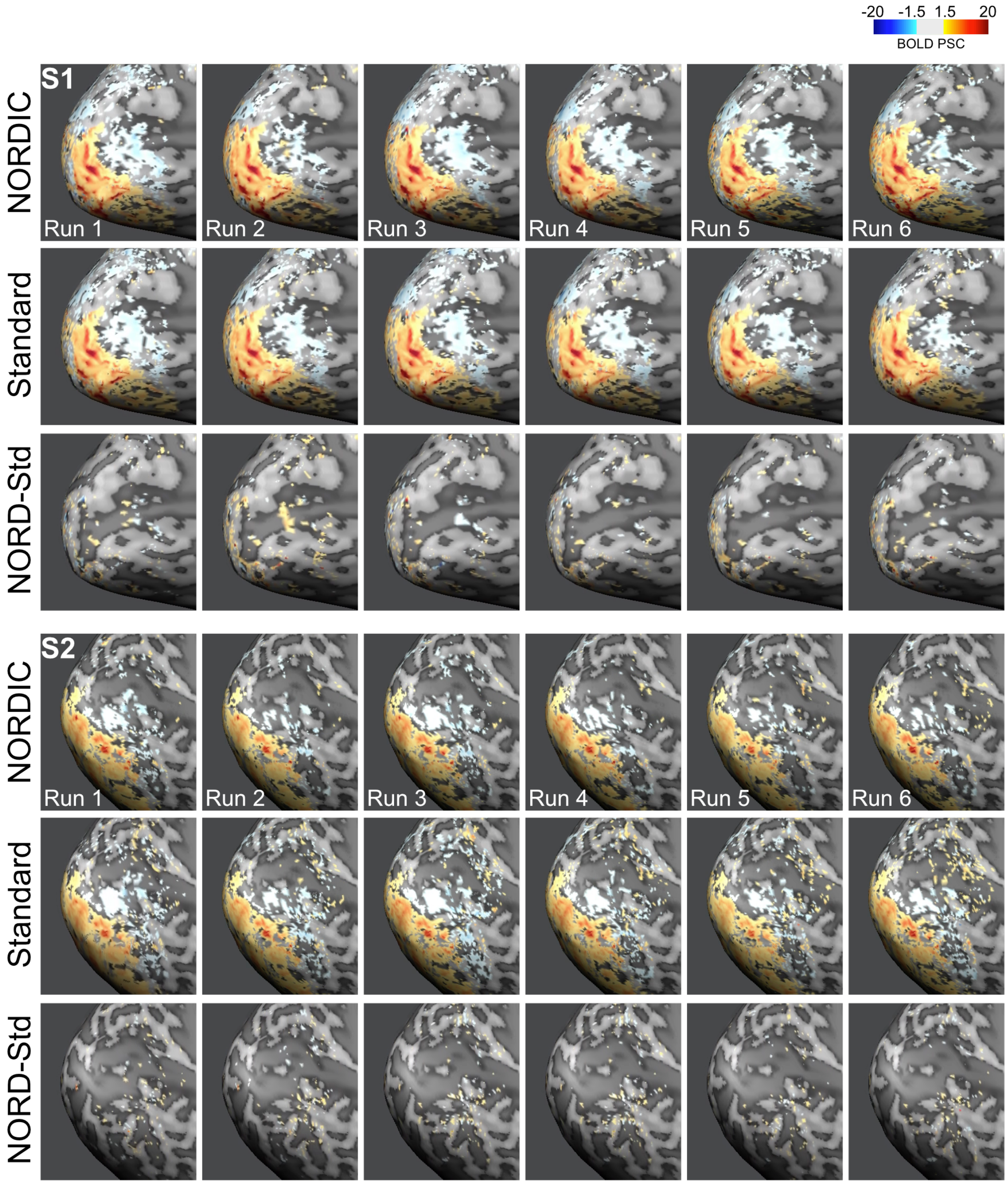

Supplementary Figure 5. Single run percent BOLD signal change (PSC) maps for two exemplar subjects. For each subject (S1 and S2 in Figure 2, main manuscript) the top row portrays the NORDIC single run BOLD PSC maps elicited by the target condition. The second row is equivalent to the first, but for Standard images. The bottom row shows the BOLD difference between NORDIC and Standard. As evident in this figure, BOLD PSC maps are highly comparable across reconstructions.

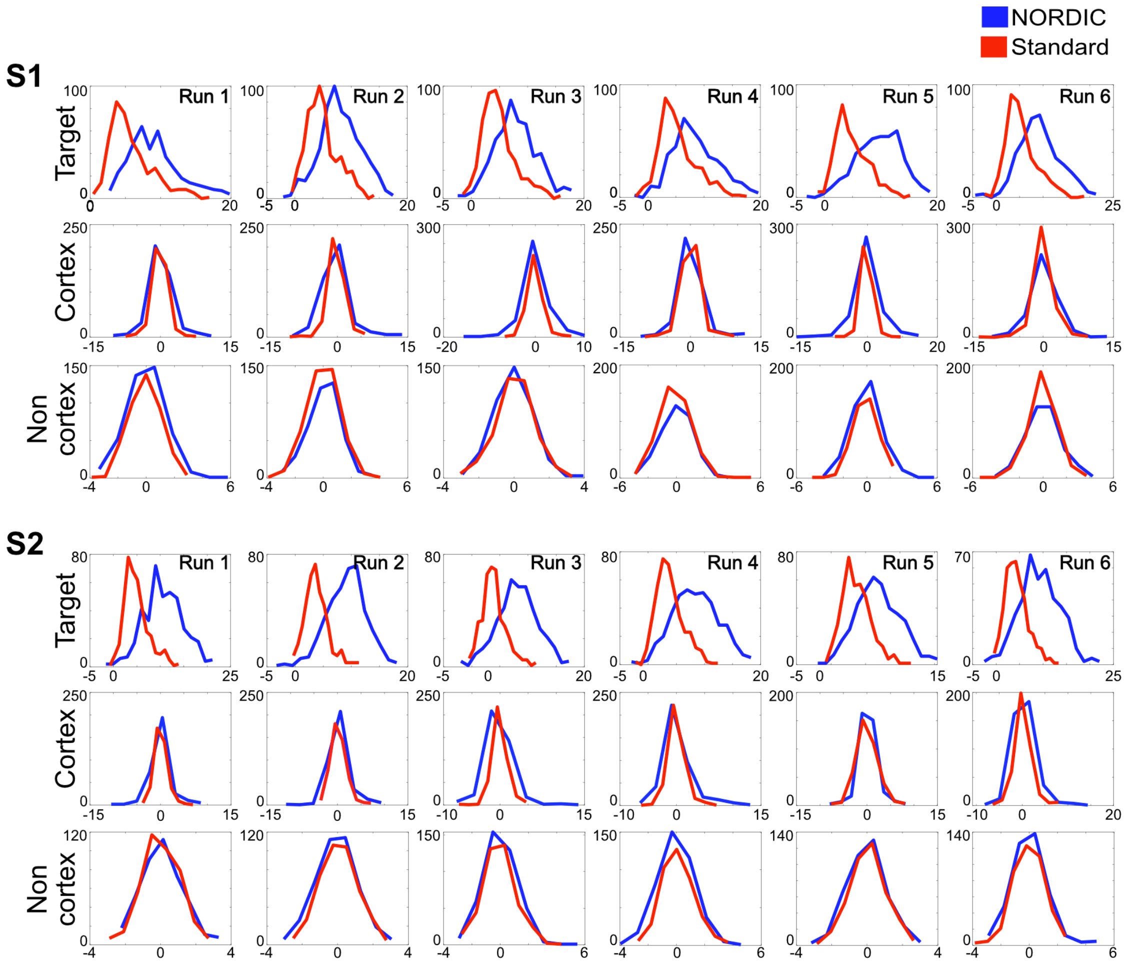

Supplementary Figure 6. NORDIC vs. Standard t-values distributions. NORDIC (blue) and Standard (red) t-value distribution for the contrast Target > 0 (see methods) for 3 ROIs. In all plots, the x axis depicts the magnitude of the t-values, while the y axis shows the voxel count. For each subject (S1 and S2 in Figure 2 main manuscript) the top row shows the histogram of the t-values for all the voxels within the target ROI. The second row shows the histogram of the t-values for a number of randomly selected voxels within the cortex not belonging to the target ROI. The third row shows the histogram of the t-values for a number of randomly selected voxels outside the cortex. For the cortex and non-cortex ROIs, the number of voxels was chosen to match that of the target ROI. The t-values distribution for NORDIC and Standard are highly non-overlapping for the target voxels. In light of the comparable PSC amplitudes across reconstructions, this set of figures indicates a substantial noise reduction for NORDIC images. The histograms for voxels within the cortex but outside the target ROI or the non-cortical voxels are essentially overlapping, demonstrating that they are not perturbed by NORDIC processing, as should be the case. Source data are provided as source Data file.

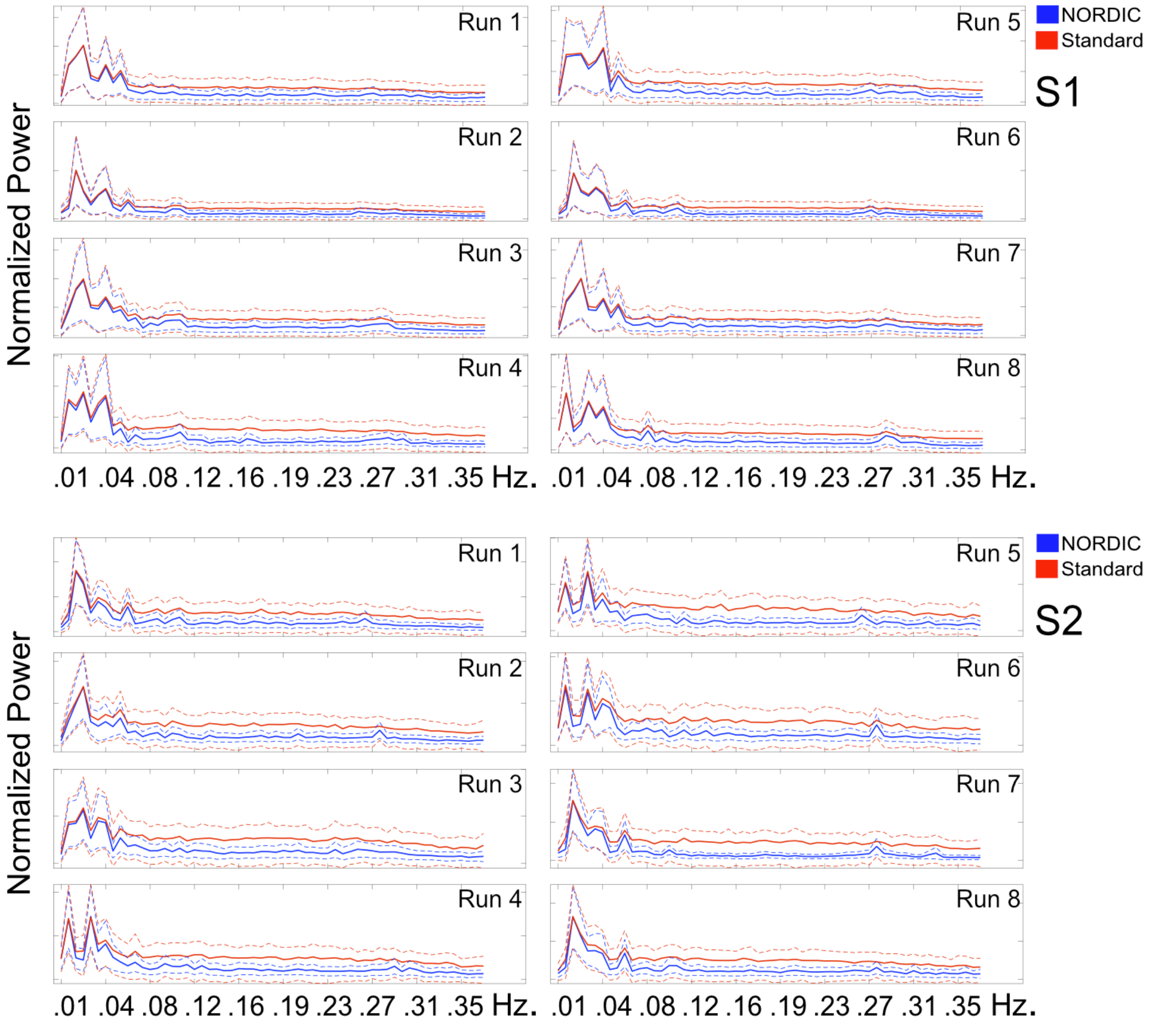

Supplementary Figure 7. NORDIC vs. Standard Fast Fourier Transform (FFT). Supplementary Figure 7 shows FFT for all runs of 2 exemplar subjects (S1 and S2 in Figure 2 of the main manuscript) for NORDIC (blue) and Standard (red) images (dotted lines show the standard deviation across voxels). FFT was computed independently per voxel within the target ROI. The magnitude output of the FFTs were then averaged across all target voxels to produce the above power spectra. The x axis portrays frequencies in Hertz. For NORDIC (but not Standard) power spectra, a peak at approximately at 0.27 Hz – representing the respiratory frequency - is visible for both subjects and most runs. These figures confirm that NORDIC denoising operates in the thermal noise regime, not impacting the peaks in the FFT spectrum but causing a general frequency independent reduction in amplitude, consistent with suppressing white noise. They also illustrate that structured physiological noise, such as respiration, becomes more easily detectable after NORDIC, as expected. Lower frequencies, representative of neuronal responses, remain largely unaffected, indicating that NORDIC denoising does not compromise neural BOLD activity. Source data are provided as source Data file.

II. Demonstrating applicability of NORDIC, across field strengths, spatial resolutions, cortical regions, stimulation and/or task paradigms, and acquisition strategies.

i) NORDIC applications at 3 Tesla:

In the manuscript, we presented only high resolution 7 Tesla fMRI data. However, vast majority of the functional imaging studies are carried out at 3 Tesla using supra-millimeter resolutions. Therefore, one of the most important indicators for the wide applicability of the NORDIC method would be to demonstrate that it can substantially improves such 3 Tesla data depending on the details of the acquisition protocol. Here, we demonstrate significant gains NORDIC imparts on the 3 Tesla data acquired with the Human Connectome Project (HCP) [1,2] protocol (Supplementary Fig. 8) and a modification of the HCP protocol to achieve higher resolution with a lower MB factor and longer TR (Supplementary Figure 9). The stimulation paradigm was same retinotopically arranged target and surround visual stimulation paradigm described in the main manuscript (see Fig. 1A in the Manuscript).

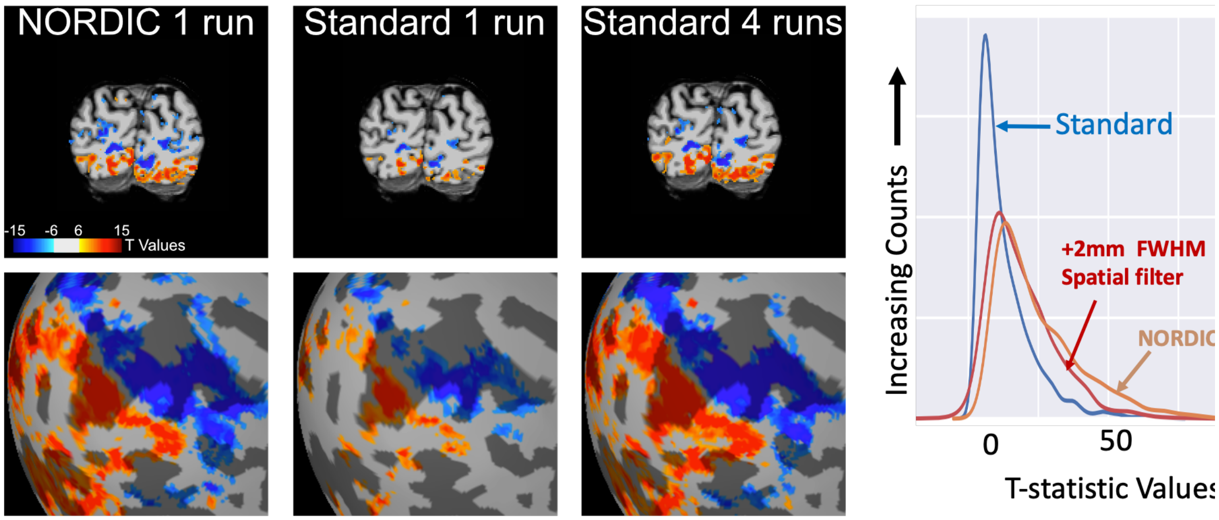

Supplementary Figure 8: The effect of NORDIC denoising on an fMRI study carried out at 3 Tesla using the standard acquisition parameters of HCP: Spatial resolution= 2 mm isotropic; TR= 800 ms; Simultaneous Multislice(SMS)/Multiband (MB) EPI acquisition with MB factor of 8. Stimulus employed was identical to that implemented in the main study described in the manuscript (see Methods), where flickering gratings for target or surround were presented in 12 second blocks interleaved by 12 second fixation periods for ~2.5 min for each run. The figure shows t-maps with t value ≥|6| for the contrast target (red) > surround (blue) in volume space for a representative slice in the visual cortex (top row), and in the inflated cortical surface (bottom row). T values >|15| appear as the same color as t=|15|. Approximately four runs with standard reconstruction (~10 min of data) are required to achieve the extent of activation comparable a single NORDIC run (~ 2.5 min of data). The right-most panel shows the t-value distribution for NORDIC, Standard reconstruction, and Standard reconstruction with 2 mm Full Width Half Maximum (FWHM) spatial smoothing, within a region of interest (ROI) created using the largest cluster from 8 runs of the data with Standard reconstruction and threshold of t=3.297, for the target *vs.* surround contrast. This ROI contained 2762 voxels. This figure demonstrates the benefit of NORDIC denoising with supra-millimeter acquisition protocols at 3 Tesla, further highlighting the wide applicability and relevance of NORDIC denoising. Source data are provided as source Data file.

As shown in Supplementary Fig. 8, the use of 4 concatenated runs representing ~10 mins of data are needed to generate an fMRI map (t-statistics map with thresholding) equivalent to that obtained from a single run (~ 2.5 min of data) using NORDIC denoising. The functional maps obtained with NORDIC using a single run is virtually identical on a pixel-by-pixel basis to that obtained with Standard 4 runs.

The t-statistics are also displayed in Supplementary Fig. 8 (right-most panel) for NORDIC and spatially smoothing with a 2 mm Full Width Half Maximum (FWHM) filter. The impact of this spatial smoothing filter on the t-statistics is similar , though not as good as to NORDIC. Unlike spatial smoothing, however, NORDIC achieves this improvement without degradation of spatial resolution (data presented in the main manuscript; also see discussion in Supplementary Fig. 11).

Looking into the future, one of the major impacts of the NORDIC approach will likely be to enable 3 Tesla studies at higher spatial resolutions relative to what has been achievable to date. We demonstrate this potential in Supplementary Fig. 9, using 1.2 mm isotropic resolution, lower MB factor 4, which may be preferred at 3T with a 32 channel coil, which results in a longer and more conventional TR.

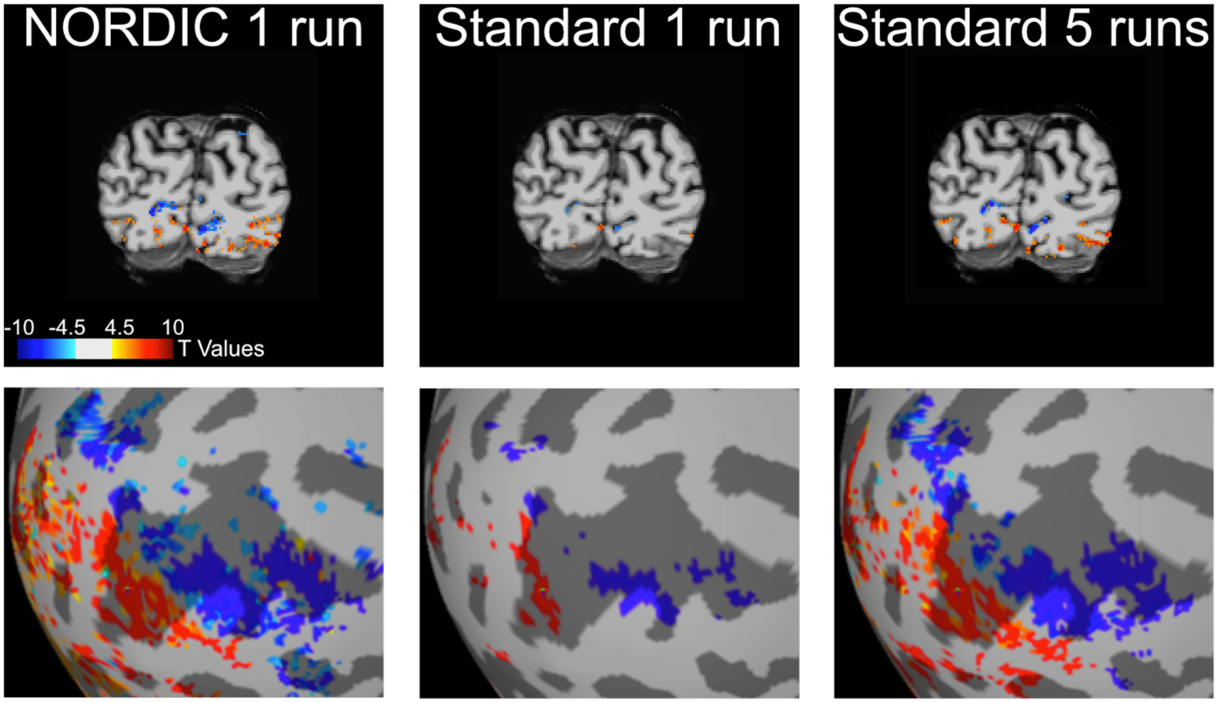

Supplementary Figure 9: The effect of NORDIC denoising on an fMRI study carried out at 3 Tesla using 1.2 mm isotropic resolution (TR 2100 ms; MB factor= 4; iPAT 2). The figure show t-maps with a t-threshold of ≥ |4.5| for the contrast target (red) > surround (blue). Because the higher resolution lengthens the echotrain length with deleterious contributions to image distortions and signal drop out, this acquisition employed parallel imaging along the phase encode direction (iPAT=2), and reduced the parallel imaging along the slice direction by using MB=4 as opposed MB=8 of the standard HCP protocol (Supplementary Fig.8). The stimulus and the presentation paradigm were the same as in Supplementary Fig.8 and identical to that implemented in the main study (see Methods), where flickering gratings for target or surround were presented for 12 seconds blocks interleaved by 12 seconds fixation periods. The figure shows t-maps for the contrast target (red) > surround (blue) in volume space, for a representative slice in the visual cortex (top row) and in inflated cortical space (bottom row). Approximately 5 Standard runs (~12 minutes of data) are required to achieve the extent of activation comparable to a single NORDIC run (~2.5 minutes of data). Given the significant differences in acquisition parameters relative to the HCP 3T data (Supplementary Fig.8), these results demonstrate the benefits of NORDIC in highly different MR context, further advocating for the generalizability of the technique.

The challenge of this higher resolution at 3 Tesla becomes evident with the functional map obtained from a single run processed with the Standard approach (Supplementary Fig. 9, middle column); this functional map is missing most of the activated territory evident in the data shown in Supplementary Fig. 8. However, the large activated territories seen in Supplementary Fig. 8 are largely recovered in NORDIC reconstruction of a single run (Supplementary Fig. 9, left column) or with Standard reconstruction after concatenating five runs, representing a much longer (~12 min) data acquisition (Supplementary Fig. 9, right most column).

ii) *EVENT RELATED Designs with CONVENTIONAL (Supra-millimeter) and HIGH (sub-millimeter) SPATIAL RESOLUTIONS and using complex COGNITIVE tasks (Face detection and gender discrimination):*

In order to demonstrate the gains achieved by NORDIC are applicable at 7T data with i) more standard (i.e. ≳ 1 mm isotropic) spatial resolutions, ii) different stimuli or tasks, iii) different paradigms, and even iv) different regions of the cortex, we turned again to the HCP acquisition protocols. The HCP had a 7 Tesla component[1,2], and this component employed 1.6 mm isotropic resolution, TR=1 s, SMS/MB acquisition with MB=5, iPAT (undersampling in phase encoded

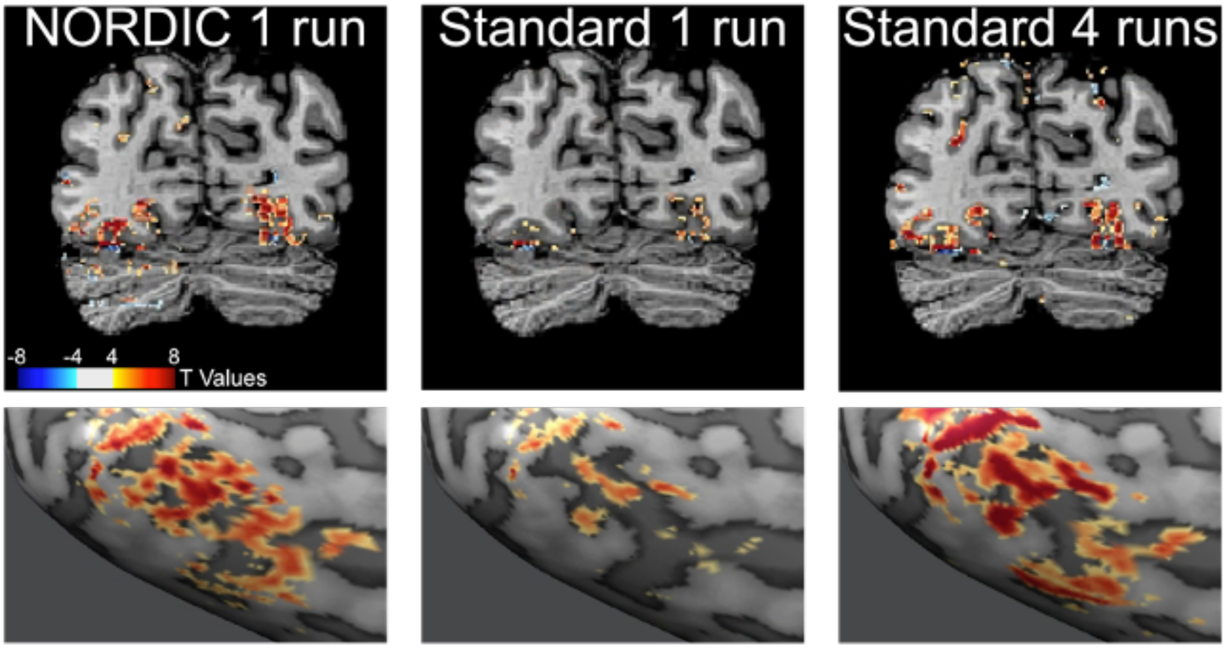

Supplementary Figure 10: The impact of NORDIC denoising on 7T data acquired with a fast event related design and standard HCP 7T fMRI protocol (i.e. 1.6 mm iso voxels; TR 1000 ms; MB 5; iPAT 2). The event related paradigm used 2 seconds stimulus-on, 2 seconds stimulus-off. A 12 s stimulus-off baseline period was also utilized at the beginning and at the end of the rapid, 2 second on-off alternating epochs. During the stimulus on periods, the participants viewed degraded images of faces (i.e. ranging from 0% to 40% image phase coherence in steps of 10% increments) while performing a face detection task. The figure shows t-maps (t ≥ |4|) for the mean activation of all conditions vs. the mean of all baseline periods when nothing was shown; the top row shows this activation map in volume space for a single slice and the lower row displays it on the inflated cortical surface in the fusiform face area. Four Standard runs (~13 minutes of data) are required to achieve the extent of activation comparable to a single NORDIC run (~3 minutes and 20 seconds of data). The HCP acquisition protocol implemented here represents a gold standard for supra-millimeter 7T fMRI; therefore, these results highlight the wide-ranging advantages conferred by NORDIC denoising across acquisition and experimental protocols.

direction) = 2. We used these acquisition parameters at 7T with a face detection paradigm where participants viewed degraded images of faces (ranging from 0% to 40% image phase coherence in steps of 10% increments) while performing a face detection task; the visual stimulation paradigm employed was a *fast event related* design where every image was presented for 2 seconds, followed by a 2 second fixation period (with 10% blank trials). The activation maps portrayed in Supplementary Fig. 10 show the T value maps with a t-threshold of $t \geq |4|$ for the mean activation to all 5 visual conditions. In this case the cortical region shown is the face fusiform area in the inferior temporal lobe.

Again, we see that single run processed with NORDIC provides a functional map that is virtually equivalent to the map obtained using multiple runs (in this case 4) concatenated and processed with Standard reconstruction algorithms, and both are far superior to what is seen with a single run processed with Standard reconstruction (Supplementary Fig.10).

Supplementary Figure. 11 compares the effect of NORDIC on the t-statistics against the effects of spatial filtering (smoothing) for an *event related paradigm* similar to that shown in Supplementary Fig. 10, but acquired with higher spatial resolution (0.8 mm isotropic) using face presentations during a gender discrimination task. Supplementary Fig.11 illustrates that applying a 0.8 mm FWHM spatial smoothing on the raw data improves the t-statistics significantly, as in the case of the 3T data shown in Supplementary Fig. 5, albeit with the effective spatial resolution degraded by approximately a factor of two in this case. NORDIC, on the other hand, performs comparable to or slightly better than spatial filtering with respect to improvements in the t-statistics (Supplementary Fig. 8), but does not degrade spatial resolution (see data presented in the main body, and also Supplementary Fig. 16).

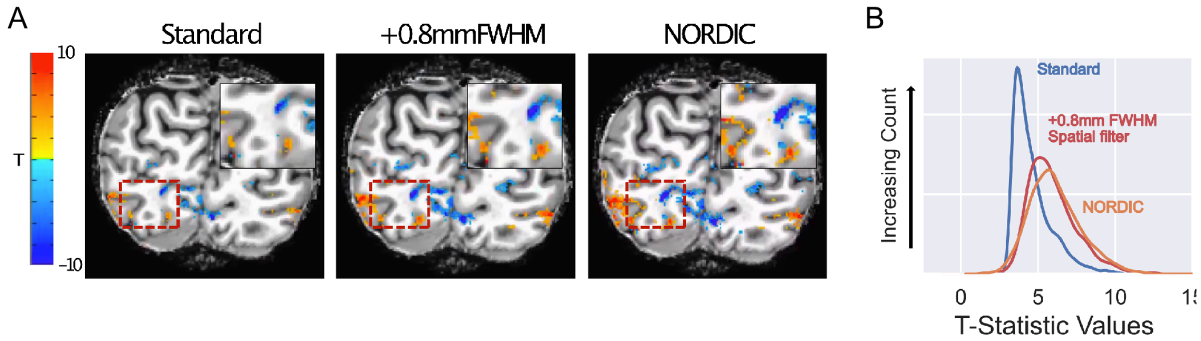

Supplementary Figure 11: T-map (panel A) and their distributions (panel B) extracted from an event related task design. Data were collected with an anterior to posterior phase encoding direction (7 Tesla, TR 1.4s, Multiband 2, iPAT 3, 6/8ths Partial Fourier, 0.8 mm isotropic resolution, TE 27.4ms, Flip Angle 78°, Bandwidth 1190Hz) and covered the occipital pole and ventral temporal cortex. Six runs of data were collected. Stimuli consisted of faces *versus* fully phase scrambled variants of the same faces. Stimuli were presented for 2 seconds, with a 2 second interstimulus interval. A. After smoothing with a 0.8mm FWHM Gaussian kernel, the maps (middle) show larger areas of activation, however, at a cost of spatial precision compared to NORDIC (right). Note that areas associated with narrow or small areas of activation are not present in smoothed data compared to NORDIC (Inset, left area). In addition, the boundary between positive and negative t-statistics is less sharp compared to NORDIC, reflecting a reduction of spatial precision due to smoothing (inset, right area). B. To summarize the effects, t-statistics for various methods were extracted using an ROI produced from 6 runs of the Standard reconstruction (blue) contrasting faces vs. scrambled. NORDIC (orange) produces t-statistic values that exceed the Standard dataset (blue). NORDIC t-statistics is more comparable to, but slightly better than that observed when the original data is spatially smoothed with a 0.8mm FWHM gaussian filter (red); however, NORDIC achieves the improvement in t-statistics without spatially blurring the image. Source data are provided as source Data file.

Spatial filtering achieves improvements in t-statistics by averaging signals over regions that are larger than those determined by the voxel dimensions set by the acquisition parameters, therefore greatly compromising the spatial resolution of the data. NORDIC instead can be thought of as having the same positive effect on the ability to detect stimulus/task induced signal changes in fMRI as spatial filtering *without* the deleterious consequences of image blurring that come with spatial filtering of images.

iii) fMRI with AUDITORY STIMULUS

As a demonstration of yet another different sensory stimulus and different cortical region, we present data obtained with auditory stimuli. The results again are comparable to those presented in the main text as well as the additional data included in this supplementary material as shown in Supplementary Figs. 8 through 11. Namely, multiple runs obtained over significantly longer data acquisition times are needed with Standard reconstruction to achieve the extent of activation comparable to a single NORDIC run.

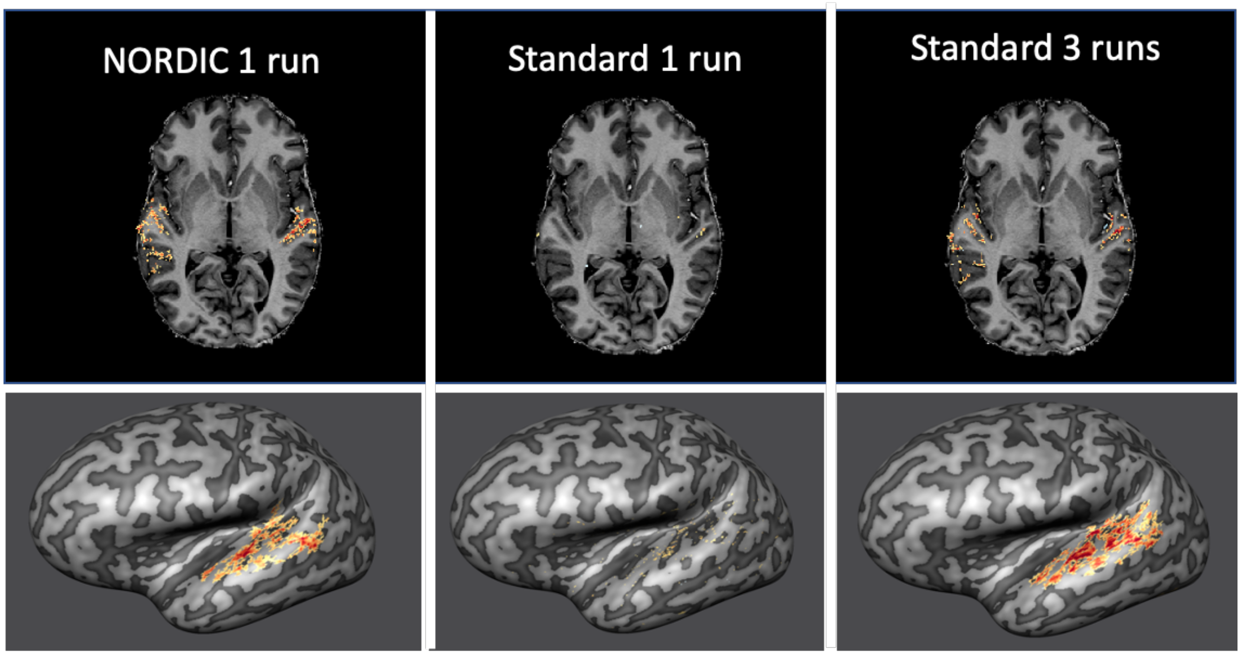

Supplementary Figure 12: The impact of NORDIC denoising on 7T data acquired in response to sound presentation (0.8 mm iso voxels; TR 1600 ms; MB 2; iPAT 3; 42 slices). Sounds (simple tones) were presented following a slow event related design (each sound lasting 1 second were presented with an average inter trial interval of 10 seconds) while participants passively listened to them. The figure shows t-maps for the main effect of sound presentation (i.e. sound vs. silence; thresholded at t > 2.7) in volume space (top row – one representative transversal slice) and in inflated cortical space (bottom row – left hemisphere). Note, 3 Standard runs (approximately 18 minutes of data) are required to achieve the extent of activation comparable to a single NORDIC run (approximately 6 minutes of data). These results highlight advantages conferred by NORDIC denoising beyond application to visual experiments.

iv) Resting state fMRI

The bulk of this work focused on task/stimulus based fMRI to demonstrate in great detail the impact of NORDIC on fMRI because in many ways task fMRI provides the ability to calculate numerous parameters, such as percent signal change, t-statistics of detection of task/stimulus induced signal changes, functional point spread function etc. that can be quantitatively evaluated

for the consequences of NORDIC processing. However, resting-state fMRI (rsfMRI) is also a frequently employed approach in neuroscience research, and is a major component of the HCP[1,2]. The NORDIC technique should work for rsfMRI. Here, we provide a preliminary demonstration of the beneficial impact of NORDIC on resting state data.

The resting state acquisition consisted of four 10 minute runs with 3T HCP acquisition parameters (Supplementary Figure 13). No stimulus presentation occurred and participants were instructed to stay still, minimize movements, and fixate on a visible crosshair.

Minimal processing steps, performed with AFNI, were applied to the Standard and NORDIC data. These included slice timing correction and motion correction to the first volume of the first run of the Standard data for both Standard and NORDIC data. Next, we regressed out the 6 estimates of motion parameters and polynomials up to 5th order. A spherical seed, with radius of 3mm was placed in the medial prefrontal cortex, corresponding to a location within the Default Mode Network. The extracted seed time course for each run was used to generate a map of Pearson's r values, corresponding to the correlation of each voxel in the brain with the seed timeseries (i.e. seed-based correlation).

These preliminary resting state NORDIC results are indeed promising. However a more in dept look at resting state acquisition and processing is required to fully appreciate the impact of NORDIC denoising on these types of acquisitions and analyses.

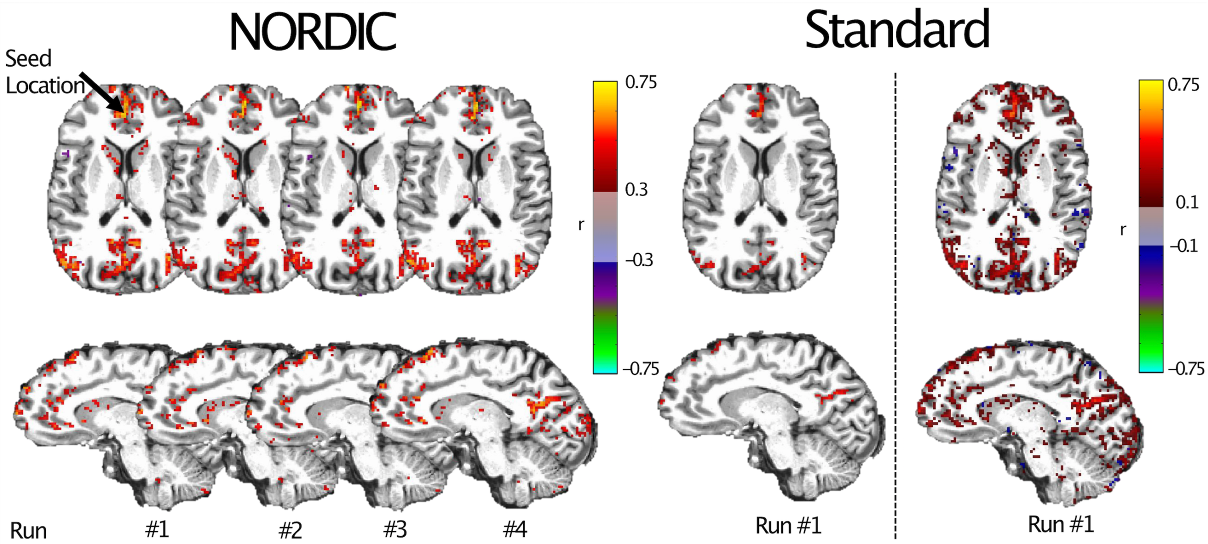

Supplementary Figure 13: Resting State fMRI. NORDIC processing benefits for resting state, seed-based connectivity. Correlation maps, with Pearson's r values are shown for 4 sequential runs of resting state data. The seed time course was extracted from the medial prefrontal cortex, corresponding to a node within the Default Mode Network. Correlation maps obtained individually from each 10 min run are highly consistent between the 4 runs of the NORDIC processed data (Left), and show cortical and subcortical correlations with r > 0.3 that are not visible in the Standard data at the same threshold (Middle). Only by reducing the standard data threshold to r>0.1 do similar patterns emerge , albeit with more punctate and scattered apparent correlations including in the white matter (Right most panel).

v) 0.5 mm isotropic data

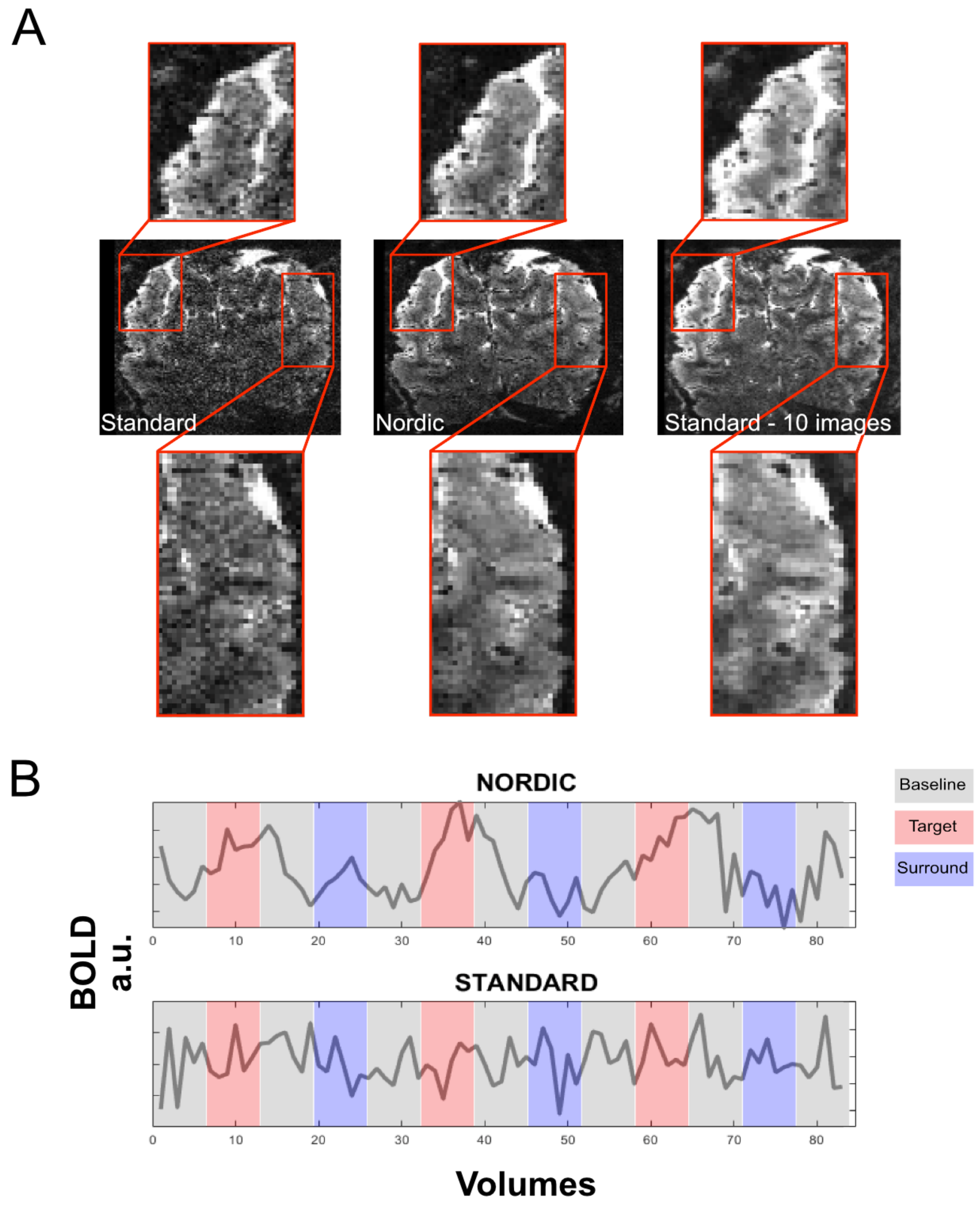

Supplementary Figure 14: Additional features of the 0.5 mm isotropic resolution data shown in Figure 6 of the main manuscript. Panel A shows a single slice from a single time point in the consecutively acquired volumes forming the fMRI time series, processed with Standrd reconstruction (left-most), NORDIC (middle) and sum of 10 images of Standard reconstruction (right most). Zoomed-in inserts demonstrate that fine features of the image observed in Standard reconstructed images are preserved in NORDIC, consistent with lack of blurring. Panel B shows the timecourse of a single medial voxel for NORDIC (top) and Standard (bottom) reconstructions froom a single run.The data were acquired using a 3D gradient echo (GE) EPI approach with a spatial resolution of 0.5 mm isotropic voxels (3D GE EPI; 40 slices; iPAT 3; TR 83 ms; Volume acqusition time 3652 ms). The total data acqusition time for a single run fMRI time series was ~ 5 minutes (i.e. 2 visual conditions - center and surround gratings – each presented 3 times for 24 seconds, and each followed by a 24 seconds fixation period. Source data are provided as source Data file.

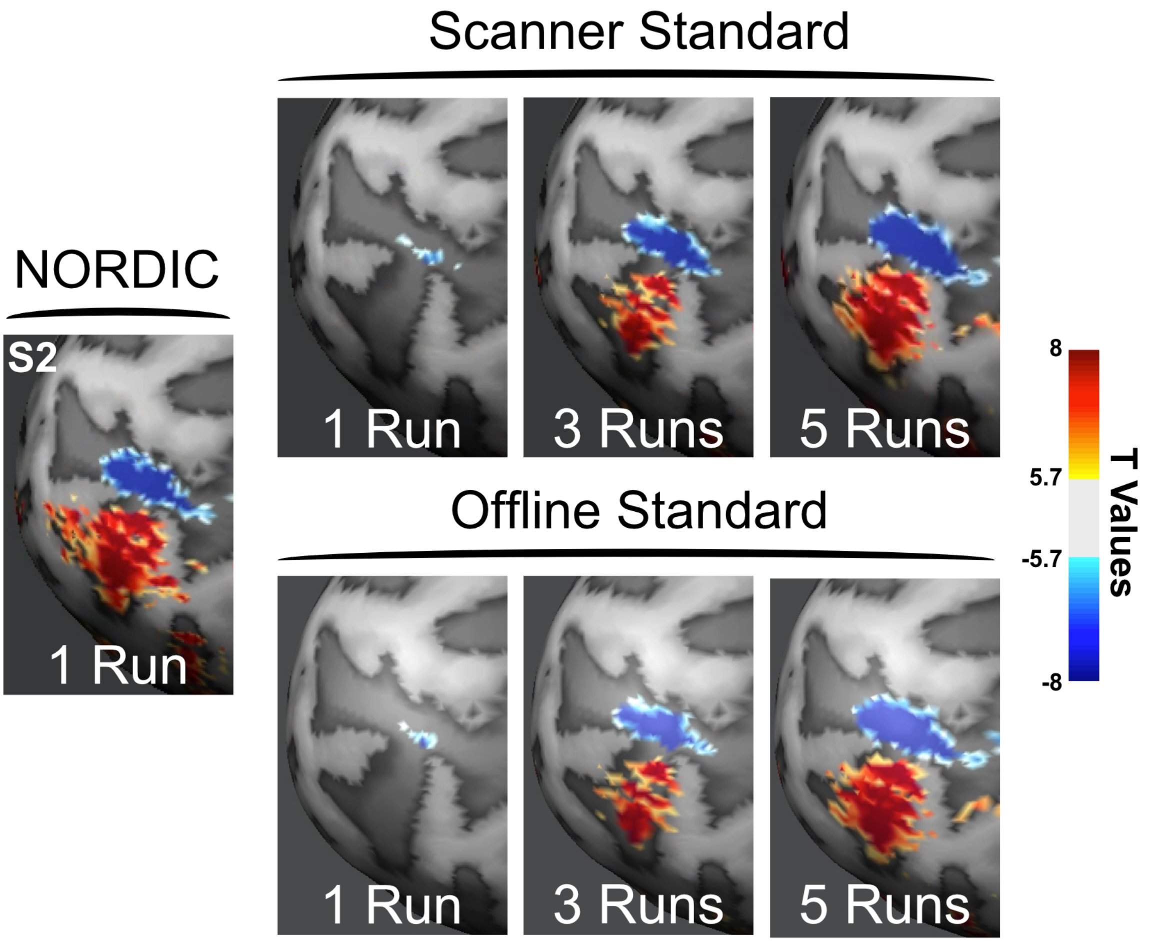

Supplemental Figure 15. Comparing NORDIC to "Offline" and "Scanner" standard**.** Top row of 3 images labelled "Scanner Standard" is a duplicate of Figure 2 for subject S2 in the main body of the paper; these functional maps, displayed for a single run and for the concatenation of 3 or 5 runs, were derived from fMRI time series of accelerated GE EPI images generated using scanner image reconstruction from the acquired k-space data. For the lower row of corresponding images labeled "Offline Standard", the same k-space data were exported offline and were processed with our offline pipeline including EPI and GRAPPA reconstructions but *without* the NORDIC denoising step. It can be seen that the two different Standard reconstructions, Scanner *versus* Offline Standard, produce virtually identical results, and for both Standards, it takes ~5 concatenated runs to achieve equivalence to NORDIC denoised single run.

All functional maps and derivative results in the main body of the paper compare NORDIC denoised results, generated by our "offline" pipeline, against those derived from fMRI time series generated using the scanner (Siemens, 7T) provided image reconstruction. It is, therefore, important to demonstrate that the scanner reconstruction produce comparable results to our offline *in the absence of* NORDIC denoising. Otherwise, the differences seen with NORDIC can be ascribed not only to the NORDIC approach but also differences in other aspects of image reconstruction. Conversely, had we only used our offline pipeline both for standard and denoised images, one can argue that our EPI and GRAPPA reconstruction may be inferior and the gains we attribute to NORDIC actually would be less had we simply used the scanner reconstruction. The results presented in this figure demonstrate that such arguments are not valid, and that without NORDIC, the scanner and our offline reconstruction produces virtually identical results.

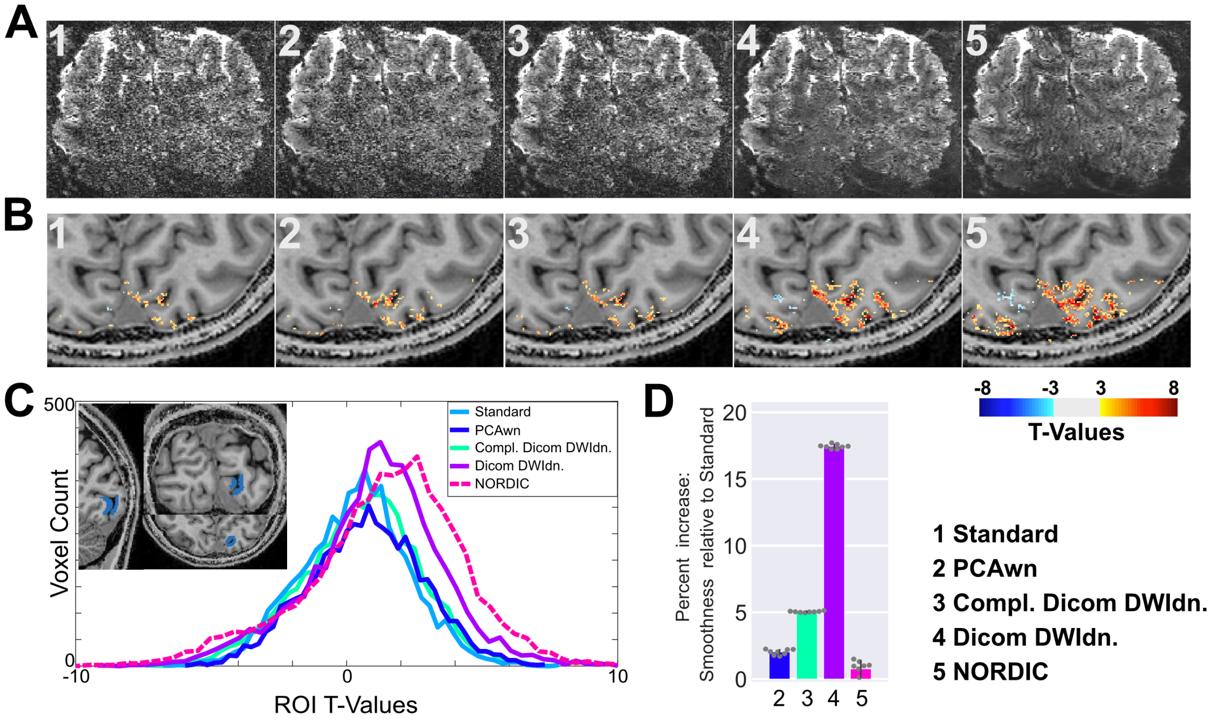

Supplemental Figure 16: Impact of different denoising strategies on 0.5 mm isotropic resolution functional data. The top row (panel A) shows a representative slice from GE EPI images acquired for different reconstructions: (1) Standard (scanner reconstruction); (2) global PCA denoising with white noise criteria (PCAwn) as described in refernce[3] to identify random noise; (3) DWI Denoise method performed on complex dicoms (Compl.DicomDWIdn); (4) DWI Denoise performed on magnitude only dicoms (Dicom DWIdn); and (5) NORDIC. The second row (panel B) shows the t-thresholded functional maps (t>|3|) computed for the contrast target > surround performed on the 8 concatenated runs for the same 5 reconstructions. Panel C shows, the t-value distributions for the same target>surround contrast for these 5 reconstructions within an ROI (shown in the top-left inlay) hand drawn on the co-registered T1wigthed images at the approximate location of the retinotopic representation of the target in V1. Panel D shows the smoothness metrics for all denoising methods in percent increase relative to the scanner Standard. For all 8 runs, smoothness was computed independently per run and estimated with a spatial autocorrelation metric using a Gaussian+monoexponential decay model as in Figure 5C in the main manuscript (see Methods for more details). Error bars represent standard errors of the mean across the 8 runs. As discussed in the results section of the main manuscript, NORDIC shows the best performance, i.e. the largest right shift for t value distribution (dashed red line) with virtually no smoothing. The t-distributions for Standard, PCAwn, and Compl. DICOM DWIdn are highly comparable; even though a relatively small improvement, accompanied by a small increase in smoothness, can be appreciated for the t-values computed for PCAwn, and Compl. DICOM DWIdn relative to Standard. The second best performance as judged only by the t-value distribution is from Dicom DWIdn; however, this is achieved with significant smoothing, which alone could be responsible for part of the improved performance. These observations are also reflected in the functional maps and the individual EPI images. *Taken together*, the metrics presented here are useful in evaluating the performance of different denoising algorithm. However, caution should be exercised in interpreting anyone metric alone, as discussed at the end of the Results section in the main manuscript. Source data are provided as source Data file.

Supplementary References

1. Ugurbil, K., Xu, J., Auerbach, E. J., Moeller, S., Vu, A. T., Duarte-Carvajalino, J. M., Lenglet, C., Wu, X., Schmitter, S., Van de Moortele, P. F., Strupp, J., Sapiro, G., De Martino, F., Wang, D., Harel, N., Garwood, M., Chen, L., Feinberg, D. A., Smith, S. M., Miller, K. L., Sotiropoulos, S. N., Jbabdi, S., Andersson, J. L., Behrens, T. E., Glasser, M. F., Van Essen, D. C., Yacoub, E. & Consortium, W. U.-M. H. *Pushing spatial and temporal resolution for functional and diffusion MRI in the Human Connectome Project*. (2013) Neuroimage **80**, 80-104.
2. Glasser, M. F., Smith, S. M., Marcus, D. S., Andersson, J. L., Auerbach, E. J., Behrens, T. E., Coalson, T. S., Harms, M. P., Jenkinson, M., Moeller, S., Robinson, E. C., Sotiropoulos, S. N., Xu, J., Yacoub, E., Ugurbil, K. & Van Essen, D. C. *The Human Connectome Project's neuroimaging approach*. (2016) Nat Neurosci **19**, 1175-1187.
3. Thomas, C. G., Harshman, R. A. & Menon, R. S. *Noise reduction in BOLD-based fMRI using component analysis*. (2002) Neuroimage **17**, 1521-1537.
