# Supplementary material for: Lowering the thermal noise barrier in functional brain mapping with magnetic resonance imaging
Source: Nat Commun. 2021 Aug 30;12:5181. doi: 10.1038/s41467-021-25431-8 (PMC8405721; doi:10.1038/s41467-021-25431-8)
Supplement: Supplementary file 2 — Reporting Summary [file 41467_2021_25431_MOESM2_ESM.pdf]

nature research

Corresponding author(s): Luca Vizioli

Last updated by author(s): Jul 21, 2021

Reporting Summary

Nature Research wishes to improve the reproducibility of the work that we publish. This form provides structure for consistency and transparency in reporting. For further information on Nature Research policies, see our Editorial Policies and the Editorial Policy Checklist.

Statistics

For all statistical analyses, confirm that the following items are present in the figure legend, table legend, main text, or Methods section.

n/a	Confirmed	
☐	☒	The exact sample size (n) for each experimental group/condition, given as a discrete number and unit of measurement
☐	☒	A statement on whether measurements were taken from distinct samples or whether the same sample was measured repeatedly
☐	☒	The statistical test(s) used AND whether they are one- or two-sided<br>*Only common tests should be described solely by name; describe more complex techniques in the Methods section.*
☐	☒	A description of all covariates tested
☐	☒	A description of any assumptions or corrections, such as tests of normality and adjustment for multiple comparisons
☐	☒	A full description of the statistical parameters including central tendency (e.g. means) or other basic estimates (e.g. regression coefficient) AND variation (e.g. standard deviation) or associated estimates of uncertainty (e.g. confidence intervals)
☐	☒	For null hypothesis testing, the test statistic (e.g. F, t, r) with confidence intervals, effect sizes, degrees of freedom and P value noted<br>*Give P values as exact values whenever suitable.*
☒	☐	For Bayesian analysis, information on the choice of priors and Markov chain Monte Carlo settings
☒	☐	For hierarchical and complex designs, identification of the appropriate level for tests and full reporting of outcomes
☐	☒	Estimates of effect sizes (e.g. Cohen's d, Pearson's r), indicating how they were calculated

Our web collection on statistics for biologists contains articles on many of the points above.

Software and code

Policy information about availability of computer code

Data collection	Data was collected using Siemens scanners operating with VE12u for 7T Terra and with VE11c for 3T Prisma
Data analysis	Data Analyses were performed using BrainVoyager 22, AFNI version 19.2.10 and Matlab R2018b, dcm2niix_afni version v1.0.20181125, distributed with AFNI (version AFNI_20.3.05)

For manuscripts utilizing custom algorithms or software that are central to the research but not yet described in published literature, software must be made available to editors and reviewers. We strongly encourage code deposition in a community repository (e.g. GitHub). See the Nature Research guidelines for submitting code & software for further information.

Data

Policy information about availability of data

All manuscripts must include a data availability statement. This statement should provide the following information, where applicable:

- Accession codes, unique identifiers, or web links for publicly available datasets
- A list of figures that have associated raw data
- A description of any restrictions on data availability

The data that support the findings of this study are available from the corresponding author upon reasonable request.

Field-specific reporting

Life sciences study design

All studies must disclose on these points even when the disclosure is negative.

Sample size	power analyses was not performed as this is a proof of concept study in which we evaluated reconstruction methods for fMRI data, where each subject represent a test re-test case
Data exclusions	one experimental run for the 0.8 mm isotropic data acquired at 7T with the visual block design was excluded due to excessive motion
Replication	For primary findings, 6-8 independent runs (each with 3 trials per condition) were used to evaluate replicability within each participant. Effects were additionally reproduced across 10 independent datasets. There were no failures to replicate.
Randomization	there were no experimental groups therefore randomization was not required
Blinding	this was not a treatment study and therefore blinding was not required. statistical tests were performed between the same data-sets analyzed with and without denoising

Reporting for specific materials, systems and methods

We require information from authors about some types of materials, experimental systems and methods used in many studies. Here, indicate whether each material, system or method listed is relevant to your study. If you are not sure if a list item applies to your research, read the appropriate section before selecting a response.

Materials & experimental systems

n/a	Involved in the study	
☒	☐	Antibodies
☒	☐	Eukaryotic cell lines
☒	☐	Palaeontology and archaeology
☒	☐	Animals and other organisms
☐	☒	Human research participants
☒	☐	Clinical data
☒	☐	Dual use research of concern

Methods

n/a	Involved in the study	
☒	☐	ChIP-seq
☒	☐	Flow cytometry
☐	☒	MRI-based neuroimaging

Human research participants

Policy information about studies involving human research participants

Population characteristics	we acquired 10 data sets on four (2 females) healthy right-handed subjects (age range: 27-33), with different stimulation paradigms, acquisition parameters and field strengths. All subjects had normal, or corrected vision and provided written informed consent
Recruitment	Recruitment was performed through the CMRR subject pool. As this study considers the effects of unstructured thermal noise, which is hardware and not population dependent, there is no impact of selection bias.
Ethics oversight	The local IRB at the University of Minnesota approved the experiments.

Note that full information on the approval of the study protocol must also be provided in the manuscript.

Magnetic resonance imaging

Experimental design

Design type	task; block and event related designs; resting state.
Design specifications	We tested the impact of NORDIC on fMRI across 4 experimental paradigms:<br>1. Block design visual stimulation<br>2. Fast event related visual stimulation design<br>3. Fast event related auditory stimulation design<br>4. Resting state.<br><br>1. Block design visual stimulation: We implemented standard block design visual stimulation paradigms for 4 acquisition types. These included the two 3T fMRI studies, the 0.8 mm isotropic resolution 7T fMRI and the 7T 0.5 mm isotropic resolution fMRI datasets. The experimental procedure consisted of a standard 12 s on, 12 s off for the 7T 0.8 mm isotropic voxel acquisitions, and for the 3T datasets, and a 24 s on, 24 s off for the 7T 0.5 mm isotropic voxel acquisitions (see Figure 1A). The difference in block length between the 2 resolutions was implemented to account the difference in

volume acquisition time between the 0.8 mm iso (i.e. volume acquisition time = 1350 ms) and the 0.5 mm iso acquisitions (i.e. volume acquisition time = 3652 ms). The stimuli consisted of a center (i.e. target) and a surround square checkerboard counterphase flickering (at 6 Hz) gratings subtending approximately 6.5 degrees of visual angle. Stimuli were centered on a background of average luminance (25.4 cd/m2, 23.5-30.1).

Each run lasted just over two and a half minutes for the 0.8 mm 7T and the 3T acquisitions (i.e. 118 volumes at 1350 ms TR) and just over 5 minutes for the 0.5 mm 7T acquisitions (85 volumes at 3654 ms volume acquisition time), beginning and ending with a 12 s or 24 s red fixation dot centered on a gray background. Within each run, each visual condition, target and surround, was presented 3 times. For the 0.5 mm iso data sets, we collected 8 experimental runs; for the 0.8 mm iso 7T and the two 3T data sets, participants underwent 8 runs, 2 of which were used to compute the region of interest and excluded from subsequent analyses. Participants were instructed to minimize movement and keep fixation locked on the center fixation dot throughout the experimental runs. For the 0.8 mm 7T acquisition on S3, run 8 had to be discarded due to excessive movement.

2. Fast event related visual design. The visual fast event related design consisted 6 runs of a face detection task, with a 2 s on, 2 s off acquisition. Each run lasted approximately 3 min and 22 s and began and ended with a 12 s fixation period. Importantly, we introduced 10% blank trials (i.e. 4 s of fixation period) randomly interspersed amongst the images, effectively jittering the ISI. Stimulus presentation was pseudorandomized across runs, with the only constraint being the non-occurrence of 2 consecutive presentations of the same phase coherence level.

We used grayscale images of faces (20 male and 20 female). We manipulated the phase coherence of each face, from 0% to 40% in steps of 10%, resulting in 200 images (5 visual conditions x 20 identities x 2 genders).

3. Fast event related auditory design. Stimuli consisted of sequences consisting of four tones. For each sequence, tones were presented for 100 ms with a 400 ms gap in between them (sequence duration 1.6 s). The sequences were presented concomitantly with the scanner noise (i.e. no silent gap for sound presentation was used) and 36 tone sequences were presented in each run, a session consisted of 10 runs of about 6 minutes each. Tone sequences were presented following a slow-event related design with an average interval of 6 TR's (ranging between 5 and 7 TR's, TR = 1.6 s)."

4. Resting state. The resting state acquisition consisted of four 10 minute runs. Data were obtained at 3T with 3T HCP acquisition parameters. No stimulus presentation occurred and participants were instructed to stay still, minimize movements and fixate on a visible crosshair.

Behavioral performance measures

the bulk of the present study focuses on neural responses to meaningless visual stimuli (i.e. flickering checkerboard gratings), where participants were required to passively observe the stimuli on-screen while in the scanner. Behavioral data were not collected

Acquisition

Imaging type(s)

functional and structural

Field strength

7 and 3 Tesla

Sequence & imaging parameters

7T Acquisition parameters.

We collected 4 variants of T2*-weighted images with different acquisition parameters, tailored to the different experimental needs. Specifically, for block design visual stimulus paradigm at 7T we collected 0.5 mm iso voxel (T2*-weighted 3D GE EPI, single slab, 40 slices, TR 83 ms, Volume Acquisition Time 3654ms, 3-fold in-plane undersampling along the phase encode direction, 6/8ths in plane Partial Fourier, 0.5 mm isotropic nominal resolution, TE 32.4ms, Flip Angle 13°, Bandwidth 820Hz). The 0.8 mm iso voxel acquisition used T2*-weighted 2D GE SMS/MB EPI, 40 slices, TR 1350 ms, Multiband factor 2, 3-fold in-plane undersampling along the phase encode direction, 6/8ths Partial Fourier, 0.8mm isotropic nominal resolution, TE 26.4ms, flip Angle 58°, Bandwidth 1190Hz . For the auditory event related design, we used a comparable submillimeter acquisition protocol (2D GE SMS/MB EPI 42 slices, TR 1600 ms, Multiband factor 2, 3-fold in-plane undersampling along the phase encode direction, 6/8ths Partial Fourier, 0.8 mm isotropic nominal resolution, TE 26.4 ms, Flip Angle 61°, Bandwidth 1190Hz)

For the visual fast event related design, we used the 7T HCP acquisition protocol (2D GE SMS/MB EPI, 85 slices TR 1s, Multiband factor 5, 2-fold in-plane undersampling along the phase encode direction, 7/8ths Partial Fourier, 1.6 mm isotropic nominal resolution, TE 22.2 ms, Flip Angle 51°, Bandwidth 1923Hz)

3T Acquisition parameters

We recorded data employed the block design visual stimulus paradigm using 2 sequences varying in resolution: Acquisition sequence 1 used the 3T HCP protocol parameters (72 slices, TR= 0.8s, Multiband= 8,no in-plane undersampling 2mm isotropic, TE =37ms, Flip Angle= 52°, Bandwidth =2290 Hz/pixel). Acquisition sequence 2 parameters were 100 slices, TR= 2.1s, Multiband= 4, in-plane undersampling factor = 2, 7/8 Partial Fourier, 1.2mm isotropic, TE= 32.6ms, Flip Angle= 78°, Bandwidth= 1595Hz/pixel

For the resting state data we used the acquisition sequence 1 detailed above (i.e. the 3T HCP protocol).

For all acquisitions, flip angles were optimized to maximize the signal across the brain for the given TR.

T1-weighted anatomical images were obtained on a 3T Siemens Magnetom Prismafit system using an MPRAGE sequence (192 slices; TR, 1900 ms; FOV, 256 x 256 mm; flip angle 9°; TE, 2.52 ms; 0.8 mm isotropic voxels) .

Area of acquisition

whole brain, occipital cortex and auditory cortex

Diffusion MRI ☐ Used ☒ Not used

Preprocessing

Preprocessing software	Data Analyses were performed using BrainVoyager 22 and AFNI version 19.2.10
Normalization	no normalization was performed
Normalization template	analyses were performed on participants brain in native space. statistical analyses were performed either at the single subject level or across BOLD responses elicited within ROIs derived for each subject. Anatomical correspondence across participants, and therefore normalization, was not required
Noise and artifact removal	Motion correction was performed using sinc interpolation relative to the first volume of the first run independently per subject
Volume censoring	no volume censoring was performed

Statistical modeling & inference

Model type and settings	to test for statistically significant differences in stimulus-evoked BOLD amplitudes and noise levels across reconstruction algorithms, we compared the ROI voxel mean percent signal change beta estimates and related t-values elicited by the target condition independently per subject. We used the 18 responses elicited by the 3 stimulus presentations within each of the 6 runs. To account for the fact that trials within each run are not independent, while the runs are, we implemented a Linear Mixed-Effect Model
Effect(s) tested	we tested differences in response amplitudes elicited by visual stimuli across reconstructions types

Specify type of analysis: ☐ Whole brain ☐ ROI-based ☒ Both

Anatomical location(s): primary visual and auditory cortices

Statistic type for inference (See Eklund et al. 2016)	paired sample t-test and (a permutation approach) 95% bootstrap confidence interval (as well as linear mixed model as explained above) were used to infer statistical significance. Neither FDR nor cluster thresholding were used to determine significance.
Correction	Bonferroni correction for multiple comparison was implemented when appropriate

Models & analysis

n/a	Involved in the study
☐	☒ Functional and/or effective connectivity
☒	☐ Graph analysis
☐	☒ Multivariate modeling or predictive analysis

Functional and/or effective connectivity	Pearson's correlation
Multivariate modeling and predictive analysis	GLM Cross validated R2 (across runs)
